# Professor Bunsen

**Published:** 1899-11

**Authors:** 


### PROFESSOR BUNSEN.

Professor Robert Wilhelm Bunsen, the eminent chemist, has recently died at Heidelberg, at the age of eighty-eight. Bunsen's greatest work dates back forty-three years, when with his master, Professor Kirchoff, of Heidelberg, he shared the honor of laying the foundations of spectroscopic astronomy, to which we owe the whole of our knowledge of the chemical constitution of the sun and stars. Newton's classical experiment of passing a beam of light through a glass prism is well known, but it led to no practical result in the advancement of science until early in the present century. In 1814 Fraunhofer, applying the same test to the light of the stars, found that different stars gave different "spectrums," as the rainbow-tinted band of light spread out by the interposition of the prism is termed. Some of these light-bands were crossed by dark lines, which were absent in others. No interpretation of the dark lines was, however, available until Kirchoff and Bunsen alighted upon a curious discovery. They found that the dark lines were identical with the bright lines produced by heating various chemical elements to incandescence and passing the light through a prism; and the inference followed that the dark lines were produced by the absorption of rays of light from those elements in the atmospheres of the sun and stars. Thus where the bright lines given by hydrogen are found to be replaced by dark lines in the spectrum of a star, hydrogen is known to be one of the constitutents of the star, and in this manner the composition of the most distant bodies in the universe is now revealed. Bunsen also made many other discoveries in chemistry, among them the charcoal pile which bears his name. Born in 1811, he had a long and brilliant university career.—*Health*, Sept., 1899.

